# Retraction: Sialoglycosylation of RBC in Visceral Leishmaniasis Leads to Enhanced Oxidative Stress, Calpain-Induced Fragmentation of Spectrin and Hemolysis

**DOI:** 10.1371/journal.pone.0224539

**Published:** 2019-10-24

**Authors:** The PLOS ONE Editors

Concerns have been raised about results reported in Figs 1F and 4C of this article [1]:

- In Fig 1F, a cell with aberrant morphology appears to be duplicated within the RBC_VL_ (sensitized) panel, and a cell cluster in this panel appears similar to a cluster in the RBC_VL_ (unsensitized) panel. The RBC_VL_ (sensitized) and RBC_VL_ (unsensitized) panels appear to include several cells and cell clusters that overlap with Fig 3B of [2]. Also, the RBC_N_ (sensitized) panel appears to overlap with data reported in Fig 3A in [2]. The images including similar elements represent different experiments in [1] and [2].
- When levels are adjusted to visualize background details, background areas within lanes 1 and 11 of Fig 4C appear more similar than would be expected for independent results, although the bands are different in the two lanes.

The authors commented that an error was made in preparing Fig 1F and they provided alternate data for the RBC_N_ (sensitized) and RBC_VL_ (both) panels. The image offered as a replacement for the RBC_VL_-sensitized panel included cells with aberrant morphologies that appear similar to cell images in the original figure and in Fig 3B of [2].

The original images supporting results in Fig 4 are no longer available.

The *PLOS ONE* Editors retract this article due to these unresolved concerns, which call into question the reliability of the results reported in Figs 1F and 4C.

CM did not agree with the retraction. The other authors did not respond or could not be reached.

Additionally, Fig 1F is excluded from this article’s [1] license because it reports material from [2], published 2007 [Elsevier Inc.], which is not offered under a CC-BY license. At the time of retraction, the article [1] was republished to note this exclusion in the Fig 1 legend and the article’s copyright statement.
